# Effects of ascorbic acid supplementation on oxidative stress markers in healthy women following a single bout of exercise

**DOI:** 10.1186/s12970-019-0269-8

**Published:** 2019-01-21

**Authors:** Manita Yimcharoen, Suwatsin Kittikunnathum, Chawannut Suknikorn, Wichuda Nak-on, Petcharee Yeethong, Tracy G. Anthony, Piyawan Bunpo

**Affiliations:** 1 Department of Medical Technology, Faculty of Associated Medical Sciences, Chiang Mai University, Chiang Mai, 50200 Thailand; 2 Department of Nutritional Sciences, Rutgers University, New Brunswick, NJ 08901 USA

**Keywords:** Exercise, Vitamin C, Oxidative stress markers, Glucose, Inflammation, Muscle damage

## Abstract

**Background:**

Ascorbic acid is a water-soluble chain breaking antioxidant. It scavenges free radicals and reactive oxygen *species* (*ROS*), which are produced during metabolic pathways. Exercise can produce an imbalance between *ROS* and antioxidants, leading to oxidative stress-related tissue damages. This study was designed to determine the effects of ascorbic acid supplementation on circulating biomarkers of oxidative stress and muscle damage following a single bout of exercise.

**Methods:**

In a crossover design with a 1 wk. wash-out period, 19 healthy women performed 30 min moderate-intensity cycling after ingesting 1000 mg of ascorbic acid (AA) or placebo. Blood samples were taken immediately before, immediately after and 30 min post-exercise to determine plasma albumin, total protein, glucose, oxidative stress and muscle damage markers.

**Results:**

Plasma albumin and total protein levels increased immediately after exercise in placebo alongside slight reductions in glucose (*p* = 0.001). These effects were absent in AA cohort. Ferric reducing ability of plasma and vitamin C levels in AA cohort significantly increased after exercise (*p* < 0.05). Superoxide dismutase activity was significantly elevated after exercise (*p* = 0.002) in placebo but not AA. Plasma malondialdehyde did not change after exercise in placebo but was significantly decreased in AA (*p* < 0.05). The exercise protocol promoted slight muscle damage, reflected in significant increases in total creatine kinase in all subjects after exercise. On the other hand, plasma C-reactive protein and lactate dehydrogenase remained unchanged.

**Conclusion:**

Supplementation with ascorbic acid prior exercise improves antioxidant power but does not prevent muscle damage.

## Background

Physical activity is an important factor in the prevention and treatment of cardiovascular diseases and regular physical exercise improves overall well-being as well as delays the ageing process [1, 2]. Exercise induces a variety of physiological changes of different magnitude and direction, depending on the characteristics of the performed exercise and on the fitness and training level of the subject. During aerobic exercise, oxygen flux can increase as much as 100-fold [3], generating reactive oxygen species (ROS) and reactive nitrogen species (RNS) [4]. If this capacity is exceeded, oxidative stress occurs, with resulting cell injury or cell death. In untrained humans and animals, one bout of moderate or high intensity exercise may cause muscle damage, followed by activation of neutrophils in response to inflammation and eventually resulting in muscle soreness [5]. Biochemical markers of muscle damage include creatine kinase [5, 6], lactate dehydrogenase (LDH) and the acute phase protein C reactive protein (CRP) in the circulation [7].

Exercise-induced production of free radicals by skeletal muscle requires the buffering capacity of an antioxidant defense system. Vitamin C or ascorbic acid (AA) is a water-soluble vitamin that is naturally present in some foods and available as a dietary supplement. Humans, unlike most animals, are unable to synthesize vitamin C endogenously, so it is an essential dietary component. The recommended daily intake for adult women is 75 mg and for adult men is 90 mg. Supplementing vitamin C in the diet to higher levels is suggested to provide antioxidant protection against oxidative stress. Antioxidant supplements are suggested to attenuate injury resulting from strenuous resistance exercise [8] and some reports claim that this approach can also improve exercise performance [9, 10]. Our previous work demonstrated that supplementation of ascorbic acid prior to exercise of moderate intensity over 3 months was associated with minor and inconsistent reductions in circulating superoxide dismutase (SOD), glutathione peroxidase (GPx), and catalase activities [11]. It has been suggested that antioxidant supplementation may only improve performance when endogenous levels are already depleted, and after reaching normal concentrations, no further benefit is seen [12]. On the other hand, recently there is a growing evidence of the negative effects of antioxidant supplementation on exercise training [13, 14].

The efficacy of vitamin C supplementation on exercise-induced oxidative stress remains unclear, some studies demonstrating no effect [15–17], and others reporting a reduction in oxidative stress markers following exercise [18, 19]. Vitamin C supplementation before exercise may also influence energy substrate selection because ascorbic acid shares the same transporters as glucose [20] and a single session of exercise increases glucose uptake by muscle cells. Therefore, this study aimed to evaluate whether ascorbic acid supplementation before exercise acutely supports antioxidant defenses and glucose metabolism and assessed its ability to prevent inflammation and muscle damage following a single bout of aerobic exercise in untrained healthy adults.

## Materials and methods

### Subjects and design

**Fig. 1:**
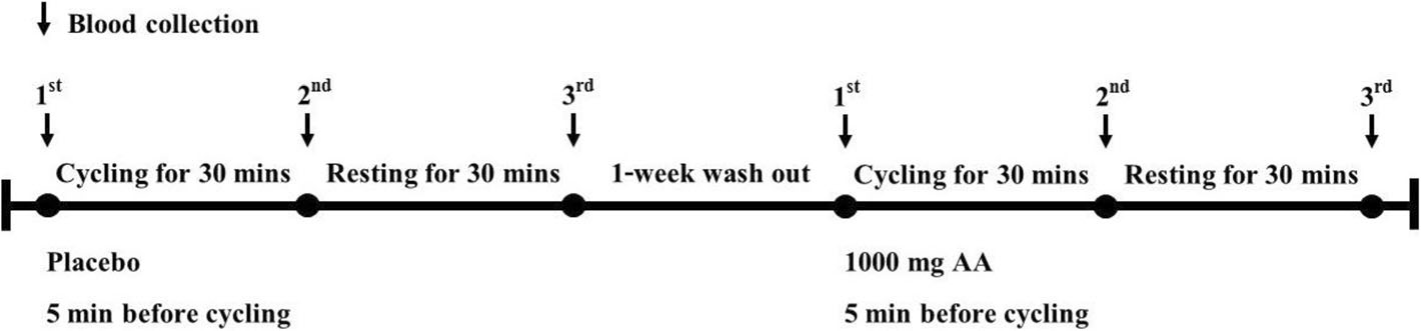
Crossover study design. Overnight fasted female subjects ingested 2 capsules containing placebo or 1000 mg of ascorbic acid (AA) 5 min prior to exercise. The exercise bout was performed indoors on a stationary bicycle for 30 min at an intensity corresponding to 65–75% of maximum heart rate. A 1-week wash out period separated the two exercise bouts

The protocol was approved by the Ethics Research Committee from Faculty of Associated Medical Sciences, Chiang Mai University. A randomized and crossover study was conducted (Fig. 1). Nineteen healthy untrained young female adults (age range 22–25 years) participated in this study after the experimental protocol was fully explained before giving their written consent to participate. Table 1 shows the main characteristics of participants in the study. The subjects were sedentary and had not participated in any regular exercise training program for at least 1 year. All subjects were nonsmokers and normotensive and had no overt history of hepatic, thyroid, renal, metabolic, cardiovascular, or pulmonary diseases, or orthopedic limitations in the exercise tests. Study participants were instructed to maintain their normal physical activity and dietary habits throughout the study. Before exercise, height and body weight were measured and body mass index (BMI) was calculated. Age-based maximum heart rate was predicted using the eq. (220-age in years). Study subjects were overnight fasted (> 8 h) before ingesting 2 gelatin capsules with water 5 min before performing 30 min/session of indoor cycling on a stationary bicycle at intensity corresponding to 65–75% of maximum heart rate. Before the first exercise bout, approximately half the subjects were randomized to receive placebo (*n* = 10) whereas the other half (*n* = 9) received 1000 mg of ascorbic acid (The Government Pharmaceutical Organization, Bangkok, Thailand). Subjects were instructed not to perform any exercise for 1 wk. (wash-out period) preceding the second bout of cycling whereupon each subject received the opposite treatment before exercise. Blood samples were collected pre-exercise, immediately after exercise and 30 min post-exercise. Heart rate (HR) was monitored continuously during the exercise to estimate energy expenditure. After exercise, all subjects were allowed to drink water according to their thirst.

**Table 1:** Characteristics of female subjects before exercise

Parameter	Placebo	Ascorbic Acid (1000 mg)
Age, years	22.4 ± 2.2	
Ethnicity/race, n	Southeast Asian (thai) 19	
Height, m	1.6 ± 0.02	
Weight, kg	53.3 ± 2.7	53.3 ± 2.6
BMI	21.2 ± 0.8	21.2 ± 0.8
Maximum heart rate (beat/min)	144 ± 2.7	144 ± 2.5
% of maximum heart rate	71 ± 1.6	71 ± 1.7
Distance, km	8.2 ± 0.2	8.1 ± 0.1
Calories, kcal	134 ± 3.3	133 ± 3.2

Values are means ± SE; n = 19 per cohort. m, meter; kg, kilogram; min, minute; km, kilometer; kcal, kilocalories

### Blood sampling and measurements

Blood samples were collected in the morning after an overnight fast of at least 8 h at the same time of exercise program. Fasting blood samples were collected into “BD VacutainerTM” test-tubes (Becton Dickinson Immunocytometry Systems-BDIS, USA) and were kept at 4 °C at all time. Plasma was obtained from the heparinized-treated blood samples within 30 min after blood collection by centrifugation (15 min, 1000×*g*, 4 °C). Plasma and erythrocytes were separated and erythrocytes were washed three times with 0.9% NaCl solution, and then hemolyzed with four volumes of cold distilled water. Prepared samples were maintained or stored (at − 20 °C) before further analyses.

### Determination of blood glucose

Plasma glucose was determined by colorimetric method [21]. The aldehyde group of glucose condenses with ortho-toluidine in glacial acetic acid. On heating, giving a green color which is measured colorimetrically at 630 nm. The intensity of color is proportion to the glucose concentration. The amount of glucose in 100 mL of blood is calculated and expressed as milligram per deciliter.

### Determination of plasma TBARS

Malondialdehyde (MDA) level was determined by thiobarbituric acid (TBA) reactive substances (TBARS) in plasma [22]. TBARS are a common way to measure lipid peroxidation products which can complement a more specific assay such as high-performance liquid chromatography. However, a variety of other compounds, such as oxidized lipids, saturated and unsaturated aldehydes, sucrose, and urea, interfere with the assay, causing overestimation of MDA concentration. In the TBARS assay, one molecule of MDA reacts with two molecules of TBA and thereby produces a pink pigment with absorption peak at 532 nm. Amplification of peroxidation during the assay is prevented by the addition of the chain-breaking antioxidant, butyryl hydroxy toluene. Results were compared using standard solution of 1,1,3,3-tetramethoxypropane and expressed as μmol per liter.

### Determination of total antioxidant status and ferric reducing ability of plasma

Antioxidant quantification was done using 2,2′ -azino-bis (3ethylbenzthiazoline-6-sulfonic acid) radical formation kinetics according to manufacturer instructions (Randox Laboratories, Ltd., Crumlin, UK). Ferric reducing ability of plasma (FRAP) assay, was performed for assessing antioxidant power. Ferric to ferrous ion reduction at low pH causes a colored ferrous-tripyridyltriazine complex to form. FRAP values are obtained by comparing the absorbance change at 593 nm in test reaction mixtures with those containing ferrous ions in known concentration. These assays were measured on Olympus AU400 auto analyzer after running control and calibration [23].

### Determination of red blood cell superoxide dismutase

The SOD activity was evaluated in the erythrocyte samples (cell lysates) by using the commercially available RANSOD Kit (Randox Laboratories, Ltd., Crumlin, UK) according to the manufacturer instructions. SOD levels were expressed as unit per gram of hemoglobin (U/g Hb). The SOD assay was measured on Olympus AU400 auto analyzer after running control and calibration.

### Determination of red blood cell glutathione peroxidase

The activity of GPx of erythrocytes lysate was evaluated with GPx detection RANSEL kit (Randox Laboratories, Ltd., Crumlin, UK) according to the manufacturer instructions, by using the appropriate control (calibrator). The GPx activity was expressed as unit per gram of hemoglobin (U/g Hb).

### Determination of erythrocyte catalase

The catalase activity in erythrocytes was assayed spectrophotometrically by monitoring the decomposition of H_2_O_2_ using the procedure of Aebi [24]. Briefly, 0.5 mL of 30 mM/L H_2_O_2_ solution in 0.95 ml of 50 mM/L phosphate buffer (pH = 7.0), 0.05 mL of 1:50 diluted erythrocyte lysates was added and the consumption of H_2_O_2_ was followed spectrophotometrically at 240 nm for 30 and 90 s at 25 °C. The molar extinction coefficient was 43.6 M^− 1^ cm^− 1^ for H_2_O_2_. Catalase activity was expressed as the unit that is defined as μM H_2_O_2_consumed/min/g hemoglobin.

### Determination of hemoglobin

Concentration of hemoglobin was determined by cyanmethemoglobin method following the method of Dacie & Lewis [25]. Hemoglobin is converted into cyanmethemoglobin by the addition of potassium cyanide and ferricyanide. The color of cyanmethemoglobin is read in a photoelectric colorimeter at 540 nm against a standard solution. Since cyanide has the maximum affinity for hemoglobin, this method estimates the total hemoglobin.

### Determination of plasma ascorbic acid

Plasma ascorbic acid was determined using ascorbic assay kit (Sigma-Aldrich*, St*. *Louis*, *MO*, USA) according to manufacturer instructions.

### Determination of plasma total protein and albumin

Total protein concentration was measured by biuret method with bovine serum protein (Sigma-Aldrich) as standard. Briefly, the peptide bonds of protein react with the copper II ions in alkaline solution to form a blue-violet complex with an absorption maximum at 545 *nm*. The color formed is proportional to the protein concentration. Albumin in plasma was determined by bromocresol green (BCG) method. The specific binding of BCG, an anionic dye, and the protein at acid pH produce a color change of the indicator from yellow–green to green–blue with the resulting shift in the absorption wavelength of the complex. The intensity of the color formed is proportional to the concentration of albumin in the sample.

### Determination of high-sensitivity C-reactive protein (hsCRP)

The plasma concentration of hsCRP was determined by commercially available kits (Randox Laboratories, Ltd., Crumlin, UK) based on an immunoturbidimetric method. Sample is reacted with a buffer and anti-CRP coated latex. The formation of the antibody-antigen complex during the reaction results in an increase in turbidity, the extent of which is measured as the amount of light absorbed at 570 nm. By constructing a standard curve from the absorbance of the standards, CRP concentration of sample can be determined.

### Determination of total creatine kinase (total CK)

The plasma creatine kinase was determined by commercially available kits (Randox Laboratories, Ltd., Crumlin, UK). Creatine kinase is made up from two genes encode highly conserved muscle (M) and brain (B) protein subunits that form three isoenzymes (MM, MB and BB). The CK-MM, MB and BB isoenzymes predominate in skeletal muscle, cardiac muscle and brain, respectively. Creatine kinase catalyses the reaction between creatinine phosphate and adenosine diphosphate (ADP) to form creatine and adenosine triphosphate (ATP). The ATP formed along with glucose is catalysed by hexokinase to form glucose-6-phosphate. The glucose-6-phosphate reduces nicotinamide adenine dinucleotide phosphate (NADP) to reduced NADP (NADPH) in the presence of glucose-6-phosphate dehydrogenase. The rate of reduction of NADP to NADPH is measured as an increase in absorbance which is proportional to the CK activity in the sample.

### Determination of creatine kinase muscle and brain isoenzymes (CK-MB)

The plasma concentration of CK-MB was analyzed according to manufacturer’s Instructions (Randox Laboratories, Ltd., Crumlin, UK). Briefly, CK-M fractions of the CK-MM and the CK-MB in the sample are completely inhibited by an anti CK-M antibody present in the reagent. Then the activity of the CK-B fraction is measured by the total CK method. The CK-MB activity is obtained by multiplying the CK-B activity by two.

### Determination of lactate dehydrogenase (LDH)

The activity of LDH in plasma was evaluated with LDH kit (Randox Laboratories, Ltd., Crumlin, UK) according to the manufacturer instructions. The LDH method measures the oxidation of L-lactate to pyruvate with simultaneous reduction of nicotinamide adenine dinucleotide (NAD). The change in absorbance at 340 nm due to the appearance of reduced NAD (NADH) is directly proportional to the LDH activity.

### Statistical analysis

At the end of the study there were *n* = 19 subjects per cohort. Results were expressed as mean ± SE. Data were processed by standard statistical software SPSS 17.0 (SPSS Software, Thailand). Two-way repeated measures analysis of variance (exercise vs. supplement) were used, differences among exercise (pre-, post- and 30 min post-exercise) and supplements (placebo and ascorbic acid) were subsequently identified using a Bonferroni *post-hoc* analysis with statistical significance of *p* < 0.05.

## Results

### Subject characteristics

Mean body weights, BMI, maximum heart rate, percent of maximum heart rate, distance and caloric expenditure were not significantly different in AA versus placebo (Table 1).

### Changes in levels of albumin, total protein and glucose

**Fig. 2:**
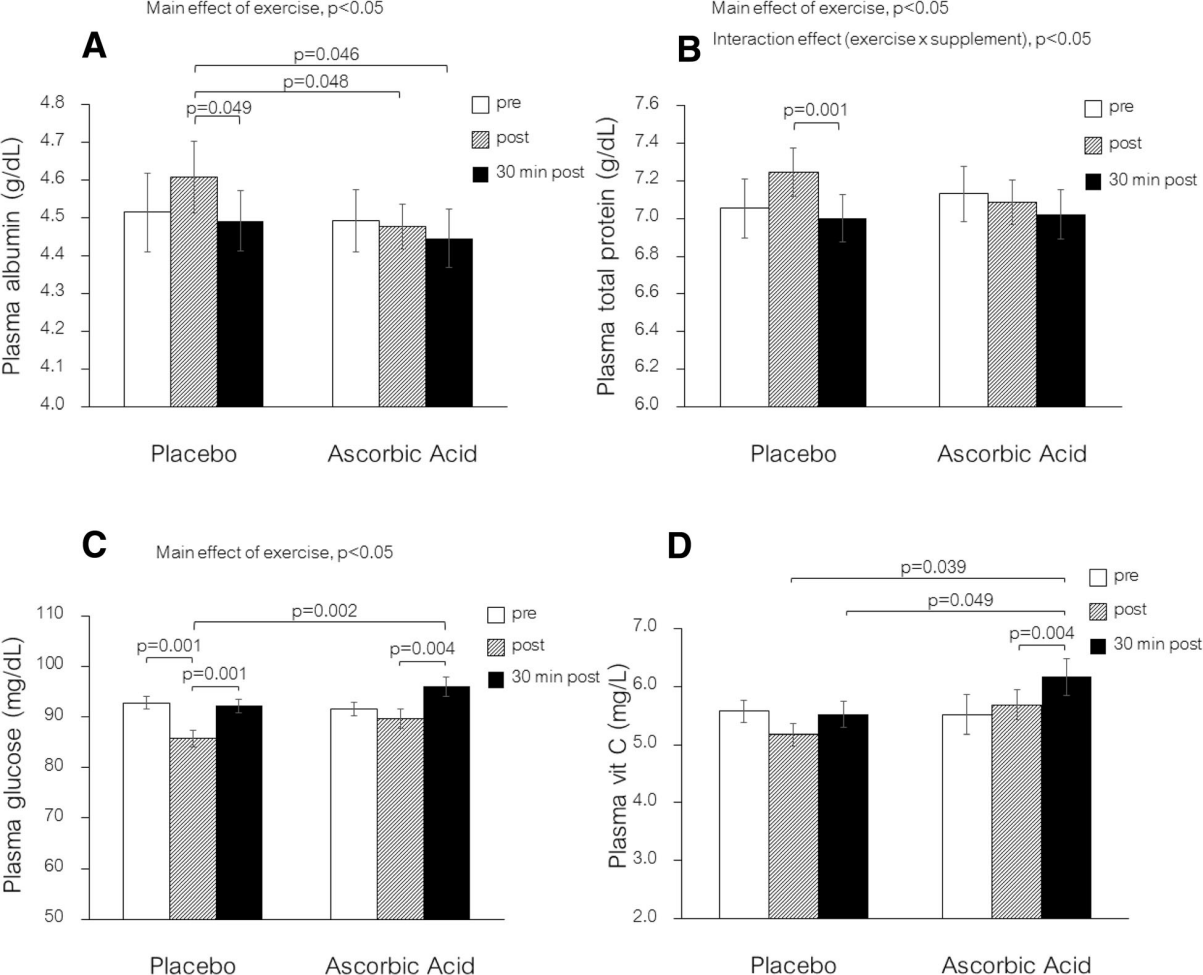
Plasma concentrations of albumin (**a**) total protein (**b**) glucose (**c**) and ascorbic acid (**d**) in females ingesting ascorbic acid or placebo before exercise. Values are means ± SE; *n* = 19 per cohort. The *p*-values indicate the results of a Bonferroni *post-hoc* analysis

There was a main effect of exercise (*p* < 0.05) for albumin and total protein levels and there was interaction between exercise and supplements for total protein (*p* < 0.05). Simple main effects analysis showed that total protein and albumin were elevated immediately after exercise and significantly (*p* = 0.049) returned to baseline at 30 min post-exercise in placebo but there was no difference between pre-, post- and 30 min post-exercise when supplemented with AA (Fig. 2a and b). There was a main effect of exercise for plasma glucose (*p* < 0.05), but no interaction between the effects of exercise and supplements. Glucose was slightly reduced immediately after exercise in placebo but remained within the normal range and returned to baseline values by 30 min post-exercise (Fig. 2c). Subjects receiving AA demonstrated a slight elevation in plasma glucose at 30 min relative to pre-exercise values but again, all values remained in the normal range.

### Changes in levels of TAS, FRAP, MDA, GPx, SOD and catalase

The acute responses in oxidative stress marker levels including TAS, FRAP, MDA, GPx, SOD and catalase to a single bout of exercise are presented in Table. 2. There was a main effect of supplement (*p* < 0.05) but no interaction between the effects of exercise and supplements for plasma MDA. No significant changes were observed in plasma MDA levels in placebo cohort, but AA cohort showed a significant reduction in MDA immediately (*p* = 0.048) and 30 min post-exercise (*p* = 0.034). There was a statistically significant interaction between the effects of exercise and supplements for FRAP (*p* < 0.05). FRAP in AA subjects was significantly (*p* = 0.002) elevated 30 min post-exercise, whereas no significant differences were found in placebo subjects. There was a main effect of exercise (*p* < 0.05) but no interaction between exercise and supplements for SOD activity. SOD activity was significantly (*p* = 0.022) elevated in placebo group at 30 min post-exercise, whereas no significant differences were found in AA group. On the other hand, the activity of GPx and catalase enzymes remained unchanged independent of treatment.

**Table 2:** Antioxidant status measured in the peripheral blood of females ingesting ascorbic acid or placebo before exercise

Test	Placebo			Ascorbic Acid (1000 mg)		
	Pre-exercise	Post-exercise	30 min post-exercise	Pre-exercise	Post-exercise	30 min post-exercise
TAS, mM Trolox equivalent	1.27 ± 0.04	1.29 ± 0.02	1.33 ± 0.02	1.28 ± 0.03	1.27 ± 0.02	1.31 ± 0.03
FRAP, μM FeSO_4_ equivalent	74.0 ± 3.29	77.0 ± 3.54	78.1 ± 3.59	73.5 ± 3.25	77.2 ± 3.15	81.2 ± 3.35 ^a, *p* = 0.002^
Plasma TBARS, μmol/g protein	0.042 ± 0.003	0.038 ± 0.003	0.044 ± 0.007	0.039 ± 0.004	0.031 ± 0.002 ^a, 0.048, b, *p* = 0.046^	0.030 ± 0.002 ^a, *p* = 0.034, b, *p* = 0.044^
Glutathione peroxidase (GPx), U/g Hb	133 ± 7.1	135 ± 7.6	128 ± 6.8	133 ± 5.3	130 ± 6.4	138 ± 7.3
Superoxide dismutase, U/g Hb	2609 ± 263	3168 ± 461	3450 ± 454^a, p = 0.022^	2605 ± 375	2726 ± 340	2794 ± 364
Catalase, U/g Hb	90 ± 11.1	90 ± 12.4	90 ± 16.2	94 ± 11.8	91 ± 16.0	92 ± 12.0

The exercise bout was performed indoors on a stationary bicycle for 30 min at intensity corresponding to 65–75% of maximum heart rate. Values are means ± SE; *n* = 19 per cohort. The *p*-values indicate the results of a Bonferroni *post-hoc* analysis. ^a^compared with pre-exercise value (within group); ^b^compared with the same time point (in between group). TAS, total antioxidant status; FRAP, ferric reducing ability of plasma; *TBARS* thiobarbituric acid reactive substances; U/g Hb, unit per gram of hemoglobin

### Changes in levels of hsCRP, LDH, total CK and CK-MB

Plasma hsCRP, LDH and CK-MB concentrations remained unchanged in subjects with or without vitamin C supplement (Table 3). There was a main effect of exercise (*p* < 0.05) but no interaction between exercise and supplements for total CK. Plasma total CK was increased immediately after exercise (*p* = 0.019) and remained high at 30 min post-exercise (*p* = 0.004) in placebo group. Total CK activity increased at 30 min post-exercise in subjects ingesting AA.

**Table 3:** Circulating markers of muscle damage and inflammation in females ingesting ascorbic acid or placebo before exercise

Test	Placebo			Ascorbic Acid (1000 mg)		
	Pre-exercise	Post-exercise	30 min post-exercise	Pre-exercise	Post-exercise	30 min post-exercise
hsCRP, mg/L	0.68 ± 0.13	0.67 ± 0.13	0.62 ± 0.12	0.57 ± 0.12	0.63 ± 0.10	0.52 ± 0.11
LDH, U/L	126 ± 5.3	124 ± 5.0	129 ± 4.8	128 ± 4.5	130 ± 4.9	132 ± 5.6
Total CK, U/L	93.5 ± 8.1	111.9 ± 10.2^a, *p* = 0.019^	111.7 ± 10.2^a, *p* = 0.004^	92.4 ± 13.7	101.0 ± 14.1	109.3 ± 14.3^a, *p* = 0.033^
CK-MB, U/L	19.7 ± 2.8	22.6 ± 3.8	15.6 ± 2.8	18.4 ± 2.6	21.3 ± 2.8	15.1 ± 2.0

The exercise bout was performed indoors on a stationary bicycle for 30 min at intensity corresponding to 65–75% of maximum heart rate. Values are means ± SE; *n* = 19 per cohort. The *p*-values indicate the results of a Bonferroni *post-hoc* analysis. ^a^compared with pre-exercise value (within group). hsCRP, high-sensitivity C-reactive protein; LDH, lactate dehydrogenase; CK, creatine kinase; CK-MB, creatine kinase-muscle and brain isoenzyme

### Ascorbic acid concentrations

There was no main effect of exercise or supplements or interaction between exercise and supplements for plasma ascorbic acid. Subjects ingesting AA demonstrated 11.8% higher plasma ascorbic acid at 30 min post-exercise as compared with their own baseline values (Fig. 2d). No differences were observed among the placebo subjects.

## Discussion

Acute exercise-induced oxidative stress is well documented over the last decade [26–28]. A single bout of physical exercise induces formation of ROS and RNS which promotes muscle damage and inflammation. Exercise has been shown to cause oxidative damage in untrained persons while in trained subjects, no such effects are observed due to an increased resistance of such persons to oxidative stress. Therefore, ROS can have beneficial and detrimental effects depends on the ROS concentration, duration of ROS exposure and training status of the individual. ROS produced during exercise lead to increased expression of antioxidants and adaptation of muscle cells. Exercise generally causes a transient increase in circulating ascorbic acid in the hours following exercise, but a decline below pre-exercise levels occurs in the days after prolonged exercise. These changes could be associated with increased exercise-induced oxidative stress [29]. Ascorbic acid is a powerful antioxidant scavenging free radicals such as the hydroxyl radical and singlet oxygen which cause tissue damage. It additionally protects vitamin E from destruction. Ascorbic acid is involved in a number of biochemical pathways that are important to exercise metabolism and the health of exercising individuals. However, there is a growing evidence of the negative effects of antioxidant supplement on exercise training [13, 14]. Therefore, the question remains whether sedentary females benefit from ascorbic acid supplementation during moderate intensity of exercise. Our understanding of the processes involved in antioxidant systems and adaptation to exercise is still limited. In the present study, we investigated the effects of pre-workout vitamin C on circulating biomarkers of oxidative stress, inflammation and muscle damage following a single bout of moderate-intensity cycling exercise. We found that ingestion of ascorbic acid immediately before exercising acutely increased antioxidant power post-exercise, evidenced by increased ferric reducing ability of plasma, decreased plasma lipid hydroperoxides and blunted superoxide dismutase enzyme activity in red blood cell lysate. Nonetheless, plasma total creatine kinase activity increased in both groups while both lactate dehydrogenase and C-reactive protein remained unchanged post-exercise, indicating that 1) ascorbic acid did not markedly alter markers of muscle damage and 2) persistent inflammation did not develop in the subjects after completing a bout of moderate intensity exercise.

During moderate exercise, glucose uptake by the working muscle rises 7 to 20 times over the basal levels [30]. This exercise induced glucose utilization without appropriate increase in endogenous glucose production coupled with delayed hepatic glucose production might explain significantly reduced levels of glucose in our exercising subjects. In addition, the metabolic fate of glucose was associated with ROS production. ROS are produced under conditions of either high or low glucose. High glucose stimulates ROS production through the activation of NADPH oxidase [31]. Wang and colleagues [32] reported that acute exposure to low glucose (40–70 mg/dL) rapidly induces endothelial dysfunction and mitochondrial oxidative stress. However, in subjects ingesting ascorbic acid, plasma glucose levels were not significantly changed after exercise and continued to elevate at 30 min post-exercise. In 1970, ascorbic acid and glucose was found to have a very similar chemical makeup and thus share transporters (GLUT1, GLUT3, and GLUT4) [33–36]. This study showed that in fasting state the ascorbic acid supplementation may interfere with glucose utilization or transportation and contribute to a lower production of ROS.

Dehydration is higher during vigorous exercise compared with mild exercise or resting state [37]. In the present study, a single bout of exercise was shown to contribute to increased levels of total protein and albumin immediately after exercise in plasma indicating dehydrated condition which was resolved after resting for 30 min. Ad libitum water consumption post-exercise was allowed and seemed to be effectively alleviated plasma volume in all subjects. Paik and colleagues [38] showed that dehydration increased oxidative DNA damage during exercise but fluid replacement with water or sports drink alleviated it equally. The increase in plasma protein and albumin following a single bout exercise was not seen in ascorbic acid supplement subjects. There was a study in rats showed that after intracerebroventricular injection of ascorbic acid resulted in a significant decrease in urinary volume and a concomitant increase in the plasma levels of vasopressin [39]. Ascorbic acid supplementation may promote adequate hydration status through antidiuretic hormone.

Plasma total CK and LDH activities were assessed as relevant blood markers of muscle damage after prolonged exercise [40]. Plasma CK activity increased immediately after exercise in placebo group whereas the increase was slightly delayed in the subjects ingesting ascorbic acid, with increased levels evident by 30 min after exercise. The current study concludes that acute supplementation with ascorbic acid does little to prevent exercise-induced muscle damage.

In this study no change of hsCRP was observed within 30 min post-exercise indicating that cycling at moderate intensity for 30 min did not induce acute inflammatory responses. However, locally induced muscle damage causing inflammation may not be easily detected systemically and hsCRP was shown to elevate at 24 to 48 h post eccentric exercise. Thus, additional later time points may be necessary to collect to capture the longer term effect of ascorbic acid supplementation following acute exercise.

Numerous studies have reported an increase in TBARS following submaximal exercise in humans, with values typically returning to baseline within one hour post-exercise [4, 41–43]. In opposition to these findings, a few studies have reported no increase in TBARS despite the use of submaximal protocols [44–46]. Our previous report showed that outdoor running for 30 min at 65–75% of maximum heart rate resulted in increased lipid peroxidation [11]. However, different types of exercise may produce different amounts of lipid peroxidation. This study showed that cycling for 30 min resulted in increased of SOD activity at 30 min post-exercise but supplementing with ascorbic acid attenuated this effect. Ascorbic acid supplementation also increased antioxidant ability as shown in FRAP assay, indicated more reducing power to protect cells from oxidizing agent. Ascorbic acid is widely distributed throughout the body with highest concentrations in the pituitary, adrenals and leukocytes. Plasma and lymphocyte ascorbic acid levels increased in nine men who completed a 21-km race [47]. However, this study showed that a bout of moderate-intensity exercise does not appears to alter blood levels of ascorbic acid. The observed increase level of ascorbic acid at 30 min post-exercise might be attributed to oral administration with ascorbic acid resulting in an increase in antioxidant capacity as shown in FRAP assay. The unaltered SOD activity after exercise in subjects receiving ascorbic acid may be explained by the increased level of ascorbic acid in blood stream which may be responsible for free radical scavenging.

There were some limitations associated with this study. First, the volunteers were healthy young adult women, therefore, the results cannot be generalized to unhealthy or older adults and the results might not be representative of the metabolic changes that occur in men as well as in the fed state. The volunteers in this study were in fasted state prior exercise. It is evidenced that pre-exercise feeding blunted signaling in skeletal muscle and adipose tissue implicated in regulating components of metabolism, including mitochondrial adaptation and substrate utilization. Thus, fasted exercise was conducted in this study. It was found that pre-exercise feeding enhanced prolonged (*P* = 0.012), but not shorter duration aerobic exercise performance (*P* = 0.687) [48]. Individuals with higher fitness levels might also react differently to the same exercise protocol. Second, the intensity of the exercise bout in this study was based on age-based prediction equation for maximal heart rate rather than from measurement obtained at maximal exercise. Multiple sample collections post-exercise is required in an attempt to provide valid ascorbic acid absorption kinetics and identification of time to peak absorption after ingestion. However, the collection time for oral intake of ascorbic acid in this study was 1 h which corresponded with the maximum concentration in plasma when orally administered at dose of 1250 mg according to the previous report [49].

## Conclusions

In summary, the present study reports that moderate-intensity of exercise by cycling increases SOD activity, this is partly due to the generation of ROS during exercise indicating the body’s ability to increase antioxidant defense capacity with acute exercise. Interestingly, moderate-intensity of exercise induces low-grade muscle damage without systemic inflammation or lipid peroxidation as measured by the formation of malondialdehyde. Ingesting ascorbic acid before a single bout of exercise increases antioxidant power in plasma and appears to mitigate the exercise-induced increase in SOD activity. Further investigation on ascorbic acid supplement during exercise is required to fully understand its interaction with other molecules.

## Abbreviations

AA: Ascorbic acid
ADP: Adenosine diphosphate
ATP: Adenosine triphosphate
BCG: Bromocresol green
BMI: Body mass index
CK: Creatine kinase
CRP: C reactive protein
FRAP: Ferric reducing ability of plasma
GLUT: Glucose transporters
GPx: Glutathione peroxidase
HR: Heart rate
LDH: Lactate dehydrogenase
MDA: Malondialdehyde
NAD: Nicotinamide adenine dinucleotide
NADH: Reduced NAD
NADP: Nicotinamide adenine dinucleotide phosphate
NADPH: reduced NADP
RNS: Reactive nitrogen species
ROS: Reactive oxygen species
TBA: Thiobarbituric acid
TBARS: Thiobarbituric acid reactive substances
U/g Hb: Unit per gram of hemoglobin
